# The association between vital signs and mortality in a retrospective cohort study of an unselected emergency department population

**DOI:** 10.1186/s13049-016-0213-8

**Published:** 2016-03-03

**Authors:** Malin Ljunggren, Maaret Castrén, Martin Nordberg, Lisa Kurland

**Affiliations:** Department of Clinical Science and Education, Södersjukhuset, Section of Emergency Medicine, Karolinska Institutet, Stockholm, Sweden; Department of Emergency Medicine and Services, Helsinki University Hospital and Helsinki University, Helsinki, Finland

**Keywords:** Emergency department, Vital signs, Mortality, Triage

## Abstract

**Background:**

Vital signs are widely used in emergency departments. Previous studies on the association between vital signs and mortality in emergency departments have been restricted to selected patient populations. We aimed to study the association of vital signs and age with 1-day mortality in patients visiting the emergency department.

**Methods:**

This retrospective cohort included patients visiting the emergency department for adults at Södersjukhuset, Sweden from 4/1/2012 to 4/30/2013. Exclusion criteria were: age < 18 years, deceased upon arrival, chief complaint circulatory or respiratory arrest, key data missing and patients who were directed to a certain fast track for conditions demanding little resources. Vital sign data was collected through the Rapid Emergency Triage and Treatment System – Adult (RETTS-A). Descriptive analyses and logistic regression models were used. The main outcome measure was 1-day mortality.

**Results:**

The 1-day mortality rate was 0.3 %. 96,512 patients met the study criteria. After adjustments of differences in the other vital signs, comorbidities, gender and age the following vital signs were independently associated with 1-day mortality: oxygen saturation, systolic blood pressure, temperature, level of consciousness, respiratory rate, pulse rate and age. The highest odds ratios was observed when comparing unresponsive to alert patients (OR 31.0, CI 16.9 to 56.8), patients ≥ 80 years to <50 years (OR 35.9, CI 10.7 to 120.2) and patients with respiratory rates <8/min to 8–25/min (OR 18.1, CI 2.1 to 155.5).

**Discussion:**

Most of the vital signs used in the ED are significantly associated with one-day mortality. The more the vital signs deviate from the normal range, the larger are the odds of mortality. We did not find a suitable way to adjust for the inherent influence the triage system and medical treatment has had on mortality.

**Conclusions:**

Most deviations of vital signs are associated with 1-day mortality. The same triage level is not associated with the same odds for death with respect to the individual vital sign. Patients that were unresponsive or had low respiratory rates or old age had the highest odds of 1-day mortality.

## Background

Vital signs are used every day in healthcare systems in a number of ways, for example, in the emergency department (ED) for diagnostics [1], as an aid in the identification of deterioration in patients, and to identify the need for intensive care unit (ICU) transfers [2, 3], and as a component in the ED triage [4, 5]. In triage, patients are prioritised according to their medical need, which has an immediate effect on their time-to-doctor. One of the main prerogatives of triage is to ensure that the sickest patient at the greatest risk of deterioration is rapidly identified. A systemic review in 2011 studied the existing evidence for the association of vital signs and presenting symptoms with mortality among patients presenting to the ED [4]. They concluded that the association between individual vital signs and mortality has rarely been studied in the ED setting and is supported by little to no evidence [4].

Previous studies on vital signs in the ED and their association to mortality have been restricted to patients who were later admitted [6, 7] and/or other selected groups of patients—for example, non-surgical patients [8, 9], trauma patients [10, 11], patients with acute coronary syndrome [12], stroke [13], infection [14], critical illness [15], or ED admission by ambulance [16]. The results have pointed in different directions. The strongest evidence is for the association with age, level of consciousness, and oxygen saturation [SpO2] (4). However, studies are lacking on vital signs in the unselected ED population in which triage is used on a daily basis.

We aimed to investigate the association between age and vital signs, measured in the triage upon arrival to the ED, and one-day mortality in an unselected population of patients. Our secondary aims were to measure the association with 30-day mortality and admission to the intensive care unit (ICU).

## Methods

### Study design and setting

This retrospective cohort study included patients who visited the adult ED at Södersjukhuset between 1 April 2012 and 30 April 2013. This study was approved by the regional ethical review board in Stockholm http://www.epn.se/en/start/ (Reference number: 2013/2301-31/4). A waiver was obtained for the requirement of written informed consent.

Södersjukhuset is a hospital in Stockholm with 649 hospital beds. It has one of the largest emergency departments in the Nordic region [17] and the largest emergency department in Stockholm, with approximately 120,000 emergency visits per year [18]. The hospital has three ICUs, one primarily for surgical and gynecological patients, one for medical patients, and one for cardiology patients requiring intensive care.

When patients arrive at the adult ED at Södersjukhuset, the patient visit is immediately registered and a quick assessment of the patient’s condition is done before the patient is directed to a section—for example, surgery, medicine, orthopaedics, or cardiology [18]. Rapid Emergency Triage and Treatment System-Adult (RETTS-A) [5], which is the most common triage system in Sweden [19], uses a combination of the patient’s presenting symptoms and signs in addition to vital sign values to determine triage priority. This triage system was the triage system in use at Södersjukhuset during the study period. At each section vital signs are measured and the patient’s presenting symptoms are matched to one of 43 Emergency Symptoms and Signs (ESS) algorithms in accordance with RETTS-A [5]. This results in one RETTS-A triage priority based on the single most deviating vital sign and one based on the severity of the emergency signs and symptoms (ESS) (5). The more urgent of the two becomes the patient’s final triage priority. The RETTS-A triage scale levels are: red, orange, yellow, green, and blue, in declining level of acuity. Patients with blue priority are directed to a fast track for non-urgent complaints and minor injuries (please see below). Patients with green priority have vital signs in, or close to, normal range and less urgent complaints than yellow, orange, and red patients.

Patients who visit the ED because of non-urgent complaints or minor injuries, who are clinically unaffected and able to move at their own capacity, are typically triaged as blue. Blue triage priority means that the patients are directed to a fast track because they are expected to require fewer resources and can be treated in a care facility without all the resources available in the ED. Receiving the triage level blue is usually done before the patient is directed to an ED section—that is, before measurement of vital signs.

### Selection of participants

The inclusion criterion was patients who visited the adult ED at Södersjukhuset between 1 April 2012 and 30 April 2013. Exclusion criteria were: age < 18 years, deceased upon arrival to the ED, data on mortality or age missing, presenting symptom registered as circulatory or respiratory arrest, and patients whose first triage priority was blue, because they are usually directed to the fast track before they have their vital signs measured. Patients unable to be followed in the Swedish population registry—for example, non-permanent residents of Sweden—were excluded due to missing data. Data that we judged incorrectly registered were set as missing in the dataset. These were 3 patients who were registered as dead for more than one day before arrival to the ED, 10 patients who had a pulse rate (PR) < 10 per minute, 30 patients who had a respiratory rate (RR) < 5 per minute and simultaneously SpO2 > 95 %, 18 patients who had a systolic blood pressure (SBP) <20 mmHg, and 47 patients who had a diastolic blood pressure (DBP) > SBP.

### Measurements

The data on vital signs was the first measured vital signs upon arrival to the ED. The following information was registered upon each patient’s arrival to the ED: presenting symptoms, SpO2 (%), RR (per minute), PR (beats per minute [bpm]), systolic blood pressure (mmHg), diastolic blood pressure (DBP, mmHg), temperature (Temp, °C) and level of consciousness according to the AVPU scale, where A is alert, V is verbal, that is, responsive to verbal stimuli, P is responsive to a pain stimuli, and U is unresponsive. Absence of previous diseases or occurrence of one or several of ten different comorbidities is registered by a nurse in triage in accordance with RETTS-A. The comorbidities are listed in Table 2B. Other diseases or conditions not directly applicable to one of the categories of comorbidities were noted with free text. Data regarding the presence of a threatened airway, oxygen use, and whether the pulse was regular or irregular was registered and incorporated in the vital sign triage prioritisation assessment, see Table 1 for information about the RETTS-A categorisation. Age, gender, time, date, admittance to in-hospital care and, if so, to which clinic, were also recorded during the ED visit. This included information regarding admittance to the ICU, which is one of the secondary outcome measures in the current study. Information regarding whether the patient had died in the month following the index ED visit, and in that case at which date, was collected through the Swedish population registry [20].

**Table 1:** Vital signs categories according to Rapid Emergency Treatment and Triage System Adult (RETTS-A) (5). Each vital sign value corresponds to a triage priority in RETTS-A (green, yellow, orange, and red). The table presents the cut-off values for each vital sign according to RETTS-A and in parentheses are the number of patients in the study population with that corresponding vital sign value in that corresponding interval

	Green	Yellow	Orange	Red
Airway	Open (*n* = 96486)			Threatened (*n* = 26)
SpO2 (%)	>95 (*n* = 79317)	90–95 (*n* = 10912)	<90 (*n* = 1848)	<90 with O_2_ (*n* = 397)
RR (min^−1^)	8–25 (86458)		26–30 (3401)	<8 (*n* = 32) or >30 (*n* = 2089)
PR (min^−1^)	50–110 (*n* = 84140)	40–49 (*n* = 601) or 111–120 (*n* = 4061)	<40 (*n* = 126) or R 121–130/IR 121–150 (*n* = 2440)	R >130 or IR > 150 (*n* = 1122)
SBP (mmHg)	≥90 (*n* = 91670)			<90 (*n* = 590)
AVPU	A (*n* = 89798)	V (*n* = 2063)	P (*n* = 478)	U (*n* = 205)
Temp (°C)	35–38.5 (*n* = 89077)	38.6–41 (*n* = 2180)	<35 (*n* = 391) or >41 (*n* = 4)	

*Abbreviations*: *SpO2* Oxygen saturation, *O*_*2*_ Oxygen, *RR* Respiratory rate, *min* minute, *PR* pulse rate, *SBP* systolic blood pressure, *AVPU* Alert, Verbal, responsive to pain, unresponsive to pain-level of consciousness scale, *Temp* temperature, *n* number

### Outcomes

The primary outcome was one-day mortality. Secondary outcomes were 30-day mortality and ICU admission. Time of admission to the ED, and the information from the Swedish population registry, were used to calculate the number of days until death for diseased patients.

### Analysis

A statistician participated in the study design and methods before conducting the analysis. We presented descriptive data on the study cohort including mean and standard deviation for baseline characteristics. Binary logistic regression models were performed to investigate the vital signs’ association to the outcomes. The crude models included one vital sign and the outcome. The adjusted models are additional binary logistic regression models that adjusted for differences in baseline characteristics. We entered all our chosen variables, i.e. vital signs, age, gender, and data on the co-morbidities described earlier into the adjusted models simultaneously. Vital signs and age were categorised for the analysis. We applied the same categorisation of vital signs as those used in RETTS-A in the statistical analyses; see Table 1 for an overview of vital sign prioritisation in RETTS-A. Age was categorised as follows: 18–49, 50–64, 65–79, and ≥80 years. Odds ratios (OR) and 95 % confidence intervals (CI) were presented. P-values < 0.05, two-sided, were considered significant. Statistical analyses were performed using the software SPSS version 22.0 (SPSS, Inc., Chicago, IL, USA).

## Results

### Characteristics of study subjects

**Fig. 1:**
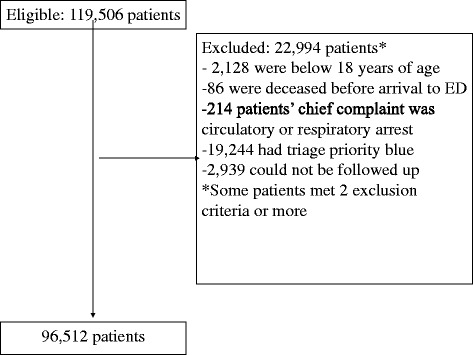
Flow chart for the derivation of the study population

There were 119,506 patients who were eligible for inclusion in the study based on the inclusion criterion. For the derivation of the study population, see Fig. 1. The one-day mortality rate of the patients who met the study criteria (*N* = 96,512) was 0.3 % during the study period. The 30-day mortality rate was 2.2 %, and the ICU admission rate was 3.1 %. The mean age for patients with green vital sign priority was 54 years old, and the mean age for the patients with red vital sign priority was 68 years. The proportion of females and males was similar for the different triage priorities. See Table 2 for further characteristics of the study population.

**Table 2:** A and B Characteristics of patients according to triage priority based on vital signs. The triage priority based on vital signs as part of the rapid emergency treatment and triage system adult (RETTS-A) is based on the vital sign which deviates most from the normal range. The patient’s final triage priority is the more urgent of either the most deviant vital sign or the patient’s presenting symptom

Characteristic	Green priority	Yellow priority	Orange priority	Red priority	Vital signs unknown	All	Missing
A							
N (%)	*N* = 69216 (71.7 %)	*N* = 13302 (13.8 %)	*N* = 6435 (6.7 %)	*N* = 3838 (4.0 %)	*N* = 3721 (3.9 %)	*N* = 96512	0
Female sex	36604 (52.9 %)	6777 (50.9 %)	3249 (50.5 %)	1907 (49.7 %)	1661 (44.6 %)	50198 (52.0 %)	0
Age in years - mean ± SD	54 ± 21	65 ± 21	67 ± 20	68 ± 20	46 ± 20	56 ± 22	0
Vital signs –mean ± SD							
SpO_2_ (%)	97.6 ± 1.5	94.5 ± 2.5	93.4 ± 7.0	92.3 ± 7.3	-	96.6 ± 3.3	4038
RR (min^−1^)	16.7 ± 2.7	17.9 ± 3.3	23.0 ± 5.5	29.3 ± 9.9	-	17.8 ± 4.6	4532
PR (min^−1^)	79.8 ± 13.8	89.7 ± 20.4	100.5 ± 26.3	111.5 ± 33.1	-	84.0 ± 19.0	4022
SBP (mmHg)	147.2 ± 26.9	145.0 ± 28.5	144.0 ± 29.3	135.7 ± 36.9	-	146.2 ± 27.9	4252
DBP (mmHg)	81.1 ± 13.7	78.9 ± 15.0	79.5 ± 16.6	75.8 ± 20.8	-	80.4 ± 14.5	4460
Temp (°C)	36.8 ± 0.5	37.1 ± 0.9	36.9 ± 1.2	37.1 ± 1.1	-	36.9 ± 0.7	4860
AVPU							3968
Alert - %	100	89.4	89.1	83.1		97.0	
Verbal - %	0	10.6	5.0	8.9		2.2	
Responsive - %	0	0	5.9	2.6		0.5	
Unresponsive - %	0	0	0	5.4		0.2	
Supplemental O2 - N (%)	785 (1.1 %)	395 (3.0 %)	508 (7.9 %)	950 (24.9 %)		2638 (2.9 %)	4038
Irregular PR-N (%)	4101 (5.9 %)	1709 (12.9 %)	1776 (27.7 %)	873 (22.8 %)		8459 (9.1 %)	4019
B							
N (%)	*N* = 69216 (71.7 %)	*N* = 13302 (13.8 %)	*N* = 6435 (6.7 %)	*N* = 3838 (4.0 %)	*N* = 3721 (3.9 %)	*N* = 96512	0
Comorbidities – N (%)							
Previously healthy	27246 (39.4 %)	2631 (19.8 %)	936 (14.5 %)	450 (11.7 %)	640 (17.2 %)	31903 (33.1 %)	
Cardiovascular disease	12489 (18.0 %)	3651 (27.4 %)	2196 (34.1 %)	1234 (32.2 %)	61 (1.6 %)	19631 (20.3 %)	
Cerebrovascular disease	2936 (4.2 %)	1040 (7.8 %)	478 (7.4 %)	291 (7.6 %)	14 (0.4 %)	4759 (4.9 %)	
Immunosuppressive disease	213 (0.3 %)	57 (0.4 %)	33 (0.5 %)	20 (0.5 %)	2 (0.1 %)	325 (0.3 %)	
Cardiac failure	1030 (1.5 %)	516 (3.9 %)	445 (6.9 %)	319 (8.3 %)	2 (0.1 %)	2312 (2.4 %)	
Kidney disease	666 (1.0 %)	216 (1.6 %)	154 (2.4 %)	109 (2.8 %)	1 (0 %)	1146 (1.2 %)	
Liver disease	258 (0.4 %)	109 (0.8 %)	35 (0.5 %)	40 (1.0 %)	0 (0 %)	442 (0.5 %)	
Lung disease	3220 (4.7 %)	1547 (11.6 %)	1159 (18.0 %)	821 (21.4 %)	34 (0.9 %)	6781 (7.0 %)	
Current malignancy	2307 (3.3 %)	765 (5.8 %)	364 (5.7 %)	265 (6.9 %)	17 (0.5 %)	3718 (3.9 %)	
Diabetes mellitus	4985 (7.2 %)	1542 (11.6 %)	800 (12.4 %)	448 (11.7 %)	36 (1.0 %)	7811 (8.1 %)	
Surgery past 6 months	496 (0.7 %)	104 (0.8 %)	42 (0.7 %)	16 (04 %)	2 (0.1 %)	660 (0.7 %)	
ED Section – N (%)							
Medicine	22216 (32.1 %)	4885 (36.7 %)	1658 (25.8 %)	813 (21.2 %)	226 (6.1 %)	29798 (30.9 %)	
Cardiology	14335 (20.7 %)	3306 (24.9 %)	3161 (49.1 %)	2186 (57.0 %)	153 (4.1 %)	23141 (24.0 %)	
Surgery	19987 (28.9 %)	3099 (23.3 %)	1112 (17.3 %)	639 (16.6 %)	316 (8.5 %)	25153 (26.1 %)	
Orthopaedia	9874 (14.3 %)	1634 (12.3 %)	409 (6.4 %)	127 (3.3 %)	626 (16.8 %)	12,670 (13.1 %)	
Other (e.g.. nurse, student)	2804 (4.1 %)	378 (2.8 %)	95 (1.5 %)	73 (1.9 %)	2400 (64.5 %)	5750 (6.0 %)	
Hospital admission	21471 (31.0 %)	6886 (51.8 %)	4371 (67.9 %)	3045 (79.3 %)	362 (9.7 %)	36135 (37.4 %)	

*Abbreviations*: *ED* emergency department, *N* number of patients

### The categorisation

The vital signs were categorised for our analysis. See Table 1 for the categorisation and the number of patients in each category. We used RETTS-A’s categorisation of vital signs. Of the included patients there were 4 patients with a temp above 41 °C, 32 patients with a RR less than 8 per minute, and 26 patients with a threatened airway.

### Main results

The majority of vital signs were associated with one-day mortality, 30-day mortality, and ICU admission. Table 3 shows the results of the analyses of the association between vital signs, age, and the outcome measures.

**Table 3:** A-C. The association of vital signs, categorised according to RETTS-A, and 1-day mortality (A), 30-day mortality (B), and ICU admission (C), expressed as OR. The tables shows both the crude univariable model and an adjusted multivariable model for each vital sign. The adjusted multivariable model was adjusted for all other vital signs, age, gender, and co-morbidities. The number of patients included in the crude models were up to 96,512 patients; in the adjusted models 90,612 were included because data on one or more variable were missing for 5,900 patients. Each vital sign value was matched to a RETTS-A priority (green, yellow, orange, red), and the patient’s presenting symptom was also matched to a RETTS-A priority. The more urgent of the two became the patient’s final priority

Vital sign	Categorization (RETTS-A priority)	1-day Crude, univariate	P for crude model	1-day Adjusted, multivariate	P for adjusted model
A. Association of vital signs and 1-day mortality					
Airway	Open (G)	1		1	
	Threatened (R)	29.3 (6.9 to 124.4)	<0.001	5.1 (0.8 to 34.1)	0.093
SpO2 (%)	>95 (G)	1		1	
	90–95 (Y)	5.8 (4.2 to 7.9)	<0.001	1.9 (1.3 to 2.7)	<0.001
	<90 (O)	24.7 (17.5 to 34.9)	<0.001	3.0 (2.0 to 4.7)	<0.001
	<90 with O2 (R)	92.2 (62.2 to 136.5)	<0.001	5.2 (3.1 to 9.0)	<0.001
RR (min^−1^)	<8 (R)	24.0 (3.3 to 177.4)	0.002	18.1 (2.1 to 155.5)	0.008
	8–25 (G)	1	<0.001	1	<0.001
	26–30 (O)	9.5 (6.7 to 13.5)	<0.001	2.4 (1.6 to 3.7)	<0.001
	>30 (R)	33.1 (25.0 to 43.8)	<0.001	4.9 (3.4 to 7.3)	<0.001
PR (min^−1^)	<40 (O)	12.0 (3.8 to 38.0)	<0.001	4.1 (1.2 to 14.4)	0.029
	40–49 (Y)	0.8 (0.1 to 5.9)	0.842	0.4 (0.1 to 3.0)	0.355
	50–110 (G)	1		1	
	111–120 (Y)	3.9 (2.7 to 5.7)	<0.001	1.9 (1.2 to 3.0)	0.009
	121–130 (O)	5.7 (3.8 to 8.5)	<0.001	1.5 (0.9 to 2.5)	0.138
	RR > 130/IR > 150 (R)	11.2 (7.3 to 17.1)	<0.001	2.8 (1.6 to 4.7)	<0.001
SBP (mmHg)	<90 (R)	28.5 (19.9 to 40.7)	<0.001	2.9 (1.8 to 4.9)	<0.001
	≥90 (G)	1		1	
AVPU	A (G)	1		1	
	V (Y)	15.9 (11.7 to 21.7)	<0.001	4.9 (3.3 to 7.1)	<0.001
	P (O)	22.3 (13.6 to 36.7)	<0.001	5.4 (2.8 to 10.5)	<0.001
	U (R)	101.7 (67.3 to 153.7)	<0.001	31.0 (16.9 to 56.8)	<0.001
Temp (°C)	<35 (O)	20.4 (12.6 to 32.9)	<0.001	4.7 (2.6 to 8.5)	<0.001
	35–38.5 (G)	1		1	
	38.6–41 (Y)	2.4 (1.4 to 4.2)	0.002	0.4 (0.2 to 0.8)	0.011
	>41 (O)	0 (0 to)	0.999	0 (0 to)	0.999
Age	<50^a^	1		1	
	50–64^a^	5.2 (2.2 to 12.3)	<0.001	7.0 (2.0 to 25.0)	0.003
	65–79^a^	19.7 (9.1 to 42.7)	<0.001	14.0 (4.1 to 47.3)	<0.001
	≥80^a^	53.3 (25.0 to 113.4)	<0.001	35.9 (10.7 to 120.2)	<0.001
B. Association of vital signs and 30-day mortality					
Vital sign	Categorization (RETTS-A priority)	30-day Crude (univariate)	P for crude	30-day Adjusted (multivariate)	P for adjusted
Airway	Open (G)	1		1	
	Threatened (R)	5.8 (1.7 to 19.3)	0.004	0.6 (0.1 to 4.1)	0.627
SpO2 (%)	>95 (G)	1		1	
	90–95 (Y)	4.8 (4.3 to 5.3)	<0.001	1.8 (1.6 to 2.0)	<0.001
	<90 (O)	15.0 (13.0 to 17.2)	<0.001	3.1 (2.6 to 3.7)	<0.001
	<90 with O2 (R)	31.4 (25.1 to 39.3)	<0.001	3.7 (2.8 to 5.0)	<0.001
RR (min^−1^)	<8 (R)	2.0 (0.3 to 14.8)	0.491	1.1 (0.1 to 9.5)	0.899
	8–25 (G)	1		1	
	26–30 (O)	6.3 (5.5 to 7.1)	<0.001	2.1 (1.8 to 2.4)	<0.001
	>30 (R)	13.6 (12.0 to 15.4)	<0.001	3.1 (2.6 to 3.6)	<0.001
PR (min^−1^)	<40 (O)	4.5 (2.4 to 8.6)	<0.001	1.9 (0.9 to 3.9)	0.085
	40–49 (Y)	1.8 (1.2 to 2.8)	0.01	1.1 (0.7 to 1.9)	0.645
	50–110 (G)	1		1	
	111–120 (Y)	2.4 (2.0 to 2.8)	<0.001	1.7 (1.4 to 2.1)	<0.001
	121–130 (O)	4.3 (3.7 to 5.0)	<0.001	2.1 (1.7 to 2.5)	<0.001
	RR > 130/IR > 150 (R)	5.5 (4.5 to 6.8)	<0.001	2.3 (1.8 to 3.1)	<0.001
SBP (mmHg)	<90 (R)	12.1 (9.9 to 14.9)	<0.001	2.9 (2.2 to 3.8)	<0.001
	≥90 (G)	1		1	
AVPU	A (G)	1	<0.001	1	
	V (Y)	8.1 (7.1 to 9.3)	<0.001	3.9 (3.3 to 4.6)	<0.001
	P (O)	13.0 (10.3 to 16.4)	<0.001	7.4 (5.3 to 10.1)	<0.001
	U (R)	28.4 (21.2 to 38.1)	<0.001	17.3 (11.2 to 26.7)	<0.001
Temp (°C)	<35 (O)	9.2 (7.1 to 12.1)	<0.001	3.2 (2.2 to 4.5)	<0.001
	35–38.5 (G)	1		1	
	38.6–41 (Y)	1.6 (1.2 to 2.0)	<0.001	0.4 (0.3 to 0.6)	<0.001
	>41 (O)	0 (0 to)	0.999	0 (0 to)	0.999
Age	<50^a^	1		1	
	50–64^a^	4.9 (3.6 to 6.6)	<0.001	2.9 (2.1 to 4.0)	<0.001
	65–79^a^	18.0 (13.7 to 23.6)	<0.001	7.5 (5.6 to 10.2)	<0.001
	≥80^a^	53.9 (41.4 to 70.2)	<0.001	21.2 (15.7 to 28.6)	<0.001
C. Association of vital signs and ICU-admission					
Vital sign	Categorization (RETTS-A priority)	ICU admission Crude (univariate)	P for crude	ICU admission Adjusted (multivariate)	P for adjusted
Airway	Open (G)	1		1	
	Threatened (R)	9.4 (3.8 to 23.5)	<0.001	1.7 (0.4 to 7.1)	0.463
SpO2 (%)	>95 (G)	1		1	
	90–95 (Y)	2.0 (1.8 to 2.2)	<0.001	1.4 (1.2 to 1.5)	<0.001
	<90 (O)	5.5 (4.7 to 6.3)	<0.001	2.6 (2.1 to 3.1)	<0.001
	<90 with O2 (R)	16.9 (13.6 to 21.1)	<0.001	4.1 (3.0 to 5.6)	<0.001
RR (min^−1^)	<8 (R)	13.0 (5.8 to 29.0)	<0.001	2.0 (0.7 to 5.2)	0.180
	8–25 (G)	1		1	
	26–30 (O)	3.1 (2.7 to 3.6)	<0.001	1.7 (1.4 to 2.0)	<0.001
	>30 (R)	7.3 (6.4 to 8.3)	<0.001	2.4 (2.0 to 2.9)	<0.001
PR (min^−1^)	<40 (O)	16.0 (10.8 to 23.7)	<0.001	17.2 (11.0 to 26.7)	<0.001
	40–49 (Y)	3.4 (2.5 to 4.6)	<0.001	2.9 (2.0 to 4.1)	<0.001
	50–110 (G)	1		1	
	111–120 (Y)	3.0 (2.7 to 3.5)	<0.001	2.3 (2.0 to 2.6)	<0.001
	121–130 (O)	4.9 (4.3 to 5.7)	<0.001	3.4 (2.9 to 4.0)	<0.001
	RR > 130/IR > 150 (R)	11.6 (10.0 to 13.4)	<0.001	6.2 (5.1 to 7.4)	<0.001
SBP (mmHg)	<90 (R)	8.0 (6.5 to 9.9)	<0.001	2.4 (1.8 to 3.2)	<0.001
	≥90 (G)	1		1	
AVPU	A (G)	1		1	
	V (Y)	8.6 (7.6 to 9.7)	<0.001	5.9 (5.1 to 6.8)	<0.001
	P (O)	24.1 (19.9 to 29.2)	<0.001	16.6 (13.2 to 20.8)	<0.001
	U (R)	64.8 (48.8 to 86.2)	<0.001	48.9 (34.4 to 69.5)	<0.001
Temp (°C)	<35 (O)	9.4 (7.4 to 12.0)	<0.001	3.2 (2.3 to 4.3)	<0.001
	35–38.5 (G)	1		1	
	38.6–41 (Y)	2.1 (1.7 to 12.5)	<0.001	0.8 (0.6 to 1.0)	0.05
	>41 (O)	11.3 (1.2 to 108.4)	0.036	2.4 (0.1 to 40.5)	0.538
Age	<50^a^	1		1	
	50–64^a^	1.6 (1.4 to 1.7)	<0.001	1.1 (0.96 to 1.2)	0.2
	65–79^a^	1.8 (1.6 to 2.0)	<0.001	0.9 (0.8 to 1.1)	0.212
	≥80^a^	1.0 (0.9 to 1.1)	0.896	0.3 (0.3 to 0.4)	<0.001

*Abbreviations*: *RETTS-A* Rapid emergency triage and treatment system adult, *SpO2* Oxygen saturation, *RR* respiratory rate, *PR* pulse rate, *SBP* Systolic blood pressure, *AVPU* Alert, Verbal, Responsive to pain, Unresponsive, *Temp* Temperature, *R* regular rhythm, *IR* irregular rhythm, *OR* odds ratio, *CI* confidence interval, *O*_*2*_ Oxygen, *G* green triage priority, *Y* yellow triage priority, *O* orange triage priority, *R* red triage priority

^a^Age is not included in the vital sign priority algorithm of RETTS-A

### Airway

In the crude models a threatened airway was associated with increased mortality. After adjustments of differences in baseline characteristics, a threatened airway was not associated with one-day mortality, 30-day mortality, and ICU admission. There was only 26 threatened airways registered, 2 patients died in the first day and another 1 within the 30 day period.

### Saturation

There are 5.2 (CI 3.1 to 9.0) times the odds of death within one day for patients with a saturation below 90 % with supplemental oxygen compared to above 95 % oxygen saturation, after adjustments of differences in baseline characteristics. In the crude models the parallel odds ratio was 92.3 (CI 62.2 to 136.5).

### Respiratory rate

Both a RR less than 8 and a RR above 30 results in a red RR according to the RETTS-A vital sign algorithm, but the results are different with respect to odds of one-day mortality so that an RR above 30 is associated with 4.9 (CI 3.4 to 7.3) times the odds of death within one day while a RR less than 8 is associated with 18.1 (CI 2.1 to 155.5) times the odds of death within one day compared to a “green” RR (8–25), after adjustments for differences in baseline characteristics. An RR less than 8 was not significantly associated with increased odds of 30-day mortality or ICU admission, but an RR over 30 remained significantly associated with increased odds of 30-day mortality as well as ICU admission.

### Pulse rate

Compared to the normal range, in the current study defined as 50–110 bpm, both a high and low PR were associated with increased one-day mortality and ICU admission. A decreased PR was not associated with increased 30-day mortality after adjustments of differences in baseline characteristics.

### Systolic blood pressure

A SBP less than 90 mmHg was associated with increased one-day and 30-day mortality and ICU admission. The OR in the adjusted model for one-day mortality was 2.9 (CI 1.8 to 4.9).

### Level of consciousness

All non-alert levels of consciousness were associated with increased odds of one-day mortality compared to alert patients; verbal patients had 4.9 (CI 3.3 to 7.1) times the odds; patients responding to a pain stimuli had 5.4 (CI 2.8 to 10.5) times the odds; and patients unresponsive to the stimuli had 31.0 (CI 16.9 to 56.8) times the odds of one-day mortality compared to alert patients after adjustments of differences in baseline characteristics. The odds of 30-day mortality and ICU admission were also increased for non-alert patients compared to alert patients.

### Temperature

Low temperatures were associated with increased odds of one-day and 30-day mortality and ICU admission. In the crude models, a temp of 38.6–41 °C was associated with increased one-day and 30-day mortality and a temp of 38.6–41 °C or above 41 °C with increased odds of ICU admission. However, after adjustments of differences in baseline characteristics, the association switched direction, and a temp of 38.6-41 °C turned out to be associated with decreased odds of one-day and 30-day mortality and ICU-admission.

### Age

An increasing age was strongly associated with higher odds for one-day and 30-day mortality in our analyses, while it was simultaneously associated with decreased odds of ICU admission.

## Discussion

In this first and largest study on a predominantly unselected population of almost 100,000 patients in the ED, we found that most of the vital sign used in the ED are significantly associated with one-day mortality, 30-day mortality, and ICU admission. The results also demonstrate that the more the vital signs deviate from the normal range, the larger the odds of mortality or ICU admission, which may be expected. Interestingly, however, the same triage level is not associated with the same odds for death with respect to the individual vital sign. As an example, a pulse rate above 130 bpm results in the same triage priority as being unconscious, that is, the highest, red, while the OR for one-day mortality is more than 30 for the unconscious patient and approximately 3 for the patient with a high pulse in the adjusted model. Moreover, in the current study we found that an increasing age was a strong predictor for both one-day and 30-day mortality but not for ICU admission.

Previous studies on selected smaller patient populations have also found these large variations in the predictive value of different vital signs [6, 7, 9]. The comparison of the predictive value of different vital signs are based on the RETTS-A categorization and cannot be automatically generalized for use in comparison between the predictive value of different vital signs in general. However, this study indicates that some vital signs predictive value for mortality is clearly underestimated in RETTS, which is the most used triage system in Sweden [19]. On the other hand, triage does not solely aim to predict mortality but to prioritize the patients with the most urgent needs highest. Patients with a decreased level of consciousness or old age had the highest odds of mortality in the current study. Age and level of consciousness upon admission to the ED have been identified as significant and relatively strong predictors of mortality in previous smaller studies on less comprehensive materials [6–9, 16, 21]. In the current study, deviations in vital signs adhering to breathing—the respiratory rate and oxygen saturation resulted in higher odds of mortality than deviations in vital signs adhering to circulatory function, that is, systolic blood pressure and pulse rate. A low or high respiratory rate [7–9] and low oxygen saturation [6, 7, 9, 16] have been identified previously as having an independent association with mortality. Results from previous studies with respect to presenting with a low systolic blood pressure are conflicting because it has been found to be associated sometimes with mortality (6–8) and other times has failed to be identified as associated with mortality [9, 16]. A low PR and a high PR are other vital sign values that have both been identified [6, 9] and have failed to be identified [7, 8, 16] as independently associated with mortality.

In the current study, a threatened airway was associated with the outcomes in the crude models but failed to reach significance when other factors were taken into consideration in the multivariate models. The reason for this may be a problem with interaction between terms. Another reason may be the small number of patients that presented with a threatened airway. Moreover, a recognized threatened airway always receive an acute intervention which may decrease the strenght of the association with the outcomes. In the current study, low temperatures were associated with high odds of mortality. It has previously been shown that sepsis patients who respond to infections with hypothermia have increased mortality compared to febrile patients [22]. Temperatures between 38.5 °C and 41 °C were associated with increased odds of death in the crude models. For some reason a high temperature was associated with decreased odds of deaths in the adjusted models. We are not sure what caused this; it may be due to an interaction between terms in the analysis. No previous study that has investigated the association between temperature and mortality in the ED setting has, to our knowledge, found that there is a significant association [8, 9].

This new knowledge of vital sign association to mortality is valuable because the unselected population in which vital signs are measured and used to determine triage priority on a daily basis is not automatically comparable to the selected populations previously studied [6–16]. This information can be used to aid in the design of a triage system that prioritises patients more accurately according to risk for mortality. This said, triage is an instrument designed to guide time-to-doctor and not to predict mortality. We believe that it is reasonable that factors other than mortality need to be taken into consideration when determining which patients need to see the doctor first, for example, risk of morbidity, severe pain, et cetera. Nonetheless, the current results provide valuable information for the future design of triage systems, as well as for ED personnel who use assessments of patient risk when making decisions regarding, for example, admittance to the hospital or level of care. The results of the current study suggests that greater value should be attributed to deviations in level of consciousness and vital signs adhering to respiratory function than what is done in the RETTS-A system. We suggest that future triage systems should also consider incorporating age, since age was an independent risk factor for mortality in the current study, and age has been associated with increased mortality in several previous studies [9, 12, 13, 16]. Patients 80 years or older had decreased odds of ICU admission despite their increased risk of one-day and 30-day mortality in the current study. We speculate that this is due to assessments of life expectancy and/or future prognosis as well as the patients’ ability to tolerate, for example, emergency surgery or intensive care. In our opinion, that a patient is not eligible for ICU-care does not mean that the patients should be given lower priority in the first assessment in the ED, that is, before the patient has even seen a physician.

### Limitations

We aimed to study the association between vital signs measured in the ED and mortality in a unselected population. We chose one of the largest EDs in the northern region of Europe as our study setting and the inclusion of patients was not limited to any one selected group or condition, but rather included all patients visiting this ED. Despite its size, this particular ED is not intended for patients with primarily psychiatric, gynecological, ear- nose- and throat, or ophthalmological conditions. We purposely restricted this study to an adult population, because vital sign values have other normal ranges for children. Hence, this is a limitation with respect to the inference of studying unselected patients. However, because the current study includes all other types of patients, we believe the results to be generalizable. The current study is to our knowledge the most comprehensive study on vital signs and mortality in an ED setting at present.

An issue to consider when interpreting the results of the current study is that we did not find a suitable way to adjust for the inherent influence the triage system and medical treatment has had on mortality. Vital signs are one of the two main components of RETTS-A. Patients presenting to the ED with the deviations in vital signs that RETTS-A recognizes as the most serious—that is, coded red—will inherently receive care first. Based on the assumption that time-to-medical interventions reduce mortality among these patients, which has been demonstrated for several conditions, for example stroke and sepsis [13, 14], triage and the prioritisation of patients may have reduced mortality for patients with extreme vital sign values. Moreover, age is not a component of the RETTS-A vital sign algorithm [5] but a factor identified in the current study and other studies, to be independently associated with mortality [9, 12, 13, 16]. Since old patients do not automatically receive a high triage priority, they do not automatically have shorter times-to-doctor. Therefore, the ORs relating to the oldest patients may be relatively high compared to the ORs relating to the patients with the largest derangements of vital signs. This is because the patients with large derangement of vital signs are set to receive faster care in RETTS-A with the imperative to decrease mortality and morbidity.

A weakness was that some clinically unaffected patients did not have their vital signs measured because they were directed to a fast track. Therefore they were excluded from the study. On the other hand, these patients were directed away from the ED partly because of the apparent non acuity of their state. Approximately 6 % of the included patients had one or more vital sign values that were either missing or registered as unable to be measured. Therefore, these patients could not be included in the adjusted models. They were, when data were available, included in the crude models. This may have affected the results in different directions, depending on the reason for the vital sign not being measured. An additional limitation of this study is that it is retrospective and single-centre.

Prospective studies are needed on vital signs’ predictive value in unselected emergency department populations. In order to study the effect of vital signs on mortality entirely without the potential effect triage has on the association, the study needs to be conducted in an ED without triage or where the existing triage decisions are not in any way linked to vital sign derangements. The ultimate categorization—that is, thresholds or cut-offs of vital signs in the unselected ED population—remains to be determined. In addition; the predictive value of combinations of vital signs as well as in combination with medical history needs to be studied.

## Conclusions

Most deviations of vital signs are associated with increased one-day mortality, 30-day mortality, and ICU admission. A decreased SpO2, a decreased or increased RR, a decreased or increased PR, a decreased SBP, a decreased level of consciousness, and a decreased body temperature as compared to the normal range were associated with increased one-day mortality in this large comprehensive study on patients seeking care at the ED. An increasing age was also associated with increased mortality. The same triage level is not associated with the same odds for death with respect to the individual vital sign. Future triage scales should consider incorporating age as a core variable and assign patients with deviations in vital sign values relating to respiratory function and level of consciousness a higher priority than those with deviations in vital signs adhering to circulation or temperature. The optimal cut-offs for vital signs in the ED setting with respect to risk remains to be determined.

## Abbreviations

AVPU: alert, responsive to verbal stimuli, responsive to pain stimuli, unresponsive
Bpm: beats per minute
CI: confidence interval
DBP: diastolic blood pressure
ED: emergency department
ESS: emergency signs and symptoms
ICU: intensive care unit
OR: odds ratio
PR: pulse rate
RETTS-A: rapid emergency triage and treatment system adult
RLS85: reaction level scale
RR: respiratory rate
SBP: systolic blood pressure
SpO2: oxygen saturation
Temp: temperature
